# *CALR* mutations in myeloproliferative neoplasms: An unfolding story

**DOI:** 10.17179/excli2020-2985

**Published:** 2020-10-26

**Authors:** Stephen E. Langabeer

**Affiliations:** 1 Cancer Molecular Diagnostics, St. James's Hospital, Dublin, Ireland

## ⁯⁯

***Dear Editor,***

The discovery of mutations in *CALR*, the gene that encodes calreticulin, in a significant proportion of patients with the myeloproliferative neoplasms (MPN) of essential thrombocythemia (ET) and primary myelofibrosis (PMF) drastically altered the molecular landscape of these diseases (Klampfl et al., 2013[4]; Nangalia et al., 2013[6]). The C-terminal of the calreticulin protein includes an endoplasmic reticulum (ER) KDEL retention signal that has a strong calcium-binding capacity thus acting as a calcium storage protein. Additionally, CALR acts as a molecular chaperone enabling glycoprotein folding in the ER. Nearly all mutations of *CALR* are either insertions, deletions or insertion and deletions (indels) that result in a loss of the terminal KDEL signal. Mutant CALR interacts with the thrombopoietin receptor MPL by a unique mechanism resulting in constitutive activation of the downstream JAK-STAT pathway and cellular transformation (Edahiro et al., 2020[2]).

Early studies suggested that *CALR* indel mutations were found inclusively in ET and PMF however expanded screening has uncovered sporadic cases exhibiting a polycythemic phenotype (Broséus et al., 2014[1]; Langabeer et al., 2017[5]). As *CALR* mutations are absent in polycythemia vera (PV; which is defined by the presence of *JAK2* mutations), this most likely represents one end of the phenotypic spectrum of *CALR*-mutated MPN. More recently, mutations in the 3' untranslated region of *CALR*, an area not covered by conventional diagnostic approaches, have been identified in patients phenotypically resembling PV (Quattrocchi et al., 2020[7]).

A variety of molecular diagnostic platforms exist to detect *CALR* mutations such as capillary electrophoresis, real-time PCR, and increasingly, next-generation sequencing. Both deep sequencing and capillary electrophoresis screening have revealed a complexity of *CALR* mutations as evidenced by co-existing, multiple indels which would not be revealed by use of real-time screening for the most common *CALR* exon 9 indels (Jeromin et al., 2016[3]; Verger et al., 2020[9]).

A further confounding observation is the low frequency of in-frame *CALR* indels that would be predicted to retain the terminal KDEL region in some patients with features hematologically suggestive of an MPN (Szuber et al., 2016[8]; Verger et al., 2020[9]). These in-frame mutations have an allelic frequency of approximately 50 %, hence suggestive of a polymorphism, and although not diagnostic of an MPN, require further functional characterization.

Taken together, these more recent findings further underscore the clonal complexity of *CALR*-mutated MPN and provide a continuing challenge for improvement of molecular diagnostics algorithms.

## Conflict of interest

The author declares no conflict of interest.
